# Foreword

**DOI:** 10.1093/annonc/mdz409

**Published:** 2019-11-18

**Authors:** D M Hyman, M Ladanyi

**Affiliations:** 1 Department of Medicine, Memorial Sloan Kettering Cancer Center, New York, NY; 2 Weill Cornell Medical College, New York, NY; 3 Department of Pathology, Memorial Sloan Kettering Cancer Center, New York, NY, USA

In this special issue of *Annals of Oncology*, leading researchers from the scientific and biomedical community provide a series of deeply researched and timely updates on the history, biological role, clinical implications and diagnostic approaches to tropomyosin receptor kinase (TRK) fusions. Collectively, their message is clear—TRK fusions play a critical oncogenic role in a broad range of cancers and their timely identification is now critical to the treatment of patients with cancers that harbour them.

In his piece, Barbacid [1] provides a personal recounting of his discovery of the first *NTRK1* fusion, isolated from a colorectal cancer specimen in 1983. Like so many practice-changing discoveries, this breakthrough was serendipitous and made alone on a Friday evening, driven by curiosity and love of science. Also, like so many discoveries, it would take decades before the clinical implications of the biologic ‘oddity’ became clear.

Next, Amatu et al. [2] provide an in-depth review of the biology of the TRK receptors, both in normal physiology and in the setting of rearrangements resulting in productive fusion proteins. In their review, they emphasise how both the limited role of TRK signalling, as well as its compartmentalisation primarily within the central nervous system, collectively permit for a favourable therapeutic index observed with select and potent TRK inhibitors, such as larotrectinib. Siena and colleagues also describe the unique pattern of TRK fusions across cancers, specifically how TRK fusions occur commonly in select rare cancers, and very rarely in most common cancers. Perhaps more than any other single factor, this unique distribution pattern may be most responsible for the extended time required for the biomedical community to translate the initial identification of TRK fusions into recent therapeutic advances.

Solomon et al. [3] follow with a comprehensive review of current diagnostic modalities available for TRK fusion detection. They describe the differing roles for pan-TRK immunohistochemistry, fluorescence *in situ* hybridisation, and perhaps most critically, broad DNA and RNA next-generation sequencing. As emphasised in their piece, there is no ‘one-size-fits-all’ approach to the pan-tumour identification of TRK fusions. Instead, testing methodologies will need to be tailored based on the underlying expected frequency of TRK fusions in each tumour type. In addition, ‘cascade testing’ should be considered, especially in cases where prior broader DNA-based profiling fails to reveal a tissue-relevant driver alteration. With the global approval of TRK inhibitors, we expect that academic and commercial laboratories will continue to mature their assays and testing algorithms over the next several years to improve the sensitivity and frequency of TRK fusion detection.

Drilon [4] next provides a timely update on the clinical development, activity, and safety of two currently Food and Drug Administration-approved TRK inhibitors, larotrectinib* and entrectinib, as well as emerging data on next-generation inhibitors, selitrectinib (BAY 2731954, LOXO-195) and repotrectinib (TPX-0005). Larotrectinib and entrectinib have both demonstrated broad pan-cancer activity, although point estimates for overall response rate and durability of response, as well as the safety profile of the agents, differ. The degree to which these efficacy and safety differences reflect the underlying target profile of these compounds versus the population enrolled in each development program is not entirely known and will require additional follow-up as well as broader clinical experience.

The effectiveness of TRK inhibitors in patients with TRK fusion-positive cancer is illustrated in two case reports. Bielack et al. [5] present a case of a paediatric patient with infantile fibrosarcoma with a rapidly progressing inoperable cervical mass unresponsive to chemotherapy. Treatment with larotrectinib resulted in rapid and dramatic tumour regression and a durable complete response that is ongoing at 16 months. In light of the marked responses with TRK inhibition and the potential for chronic treatment in children, this case highlights the need to better understand the long-term consequences of such treatment, both in terms of safety and normal development. Finally, O’Reilly and Hechtman [6] present us with a case report of a patient with TRK fusion-positive pancreatic cancer treated serially with both first- and next-generation TRK inhibitors. This case highlights not only the potential for TRK inhibitors to offer benefit even to the most refractory cancers, but also the complexity of managing acquired resistance driven by both on- and off-target mechanisms.

As Guest Editors for this Special Supplement, we hope you will find this set of articles useful in providing background for future discussions that will shape further clinical advances in this subset of patients with cancer and inform approaches to other cancers that present similar diagnostic challenges. 

## Funding

This paper was published as part of a supplement financially supported by Bayer AG and Loxo Oncology, Inc., a wholly owned subsidiary of Eli Lilly and Company.

## Disclosure

DMH discloses consulting or advisory roles with AstraZeneca, Bayer, Boehringer Ingelheim, Chugai Pharma, CytomX Therapeutics, Genentech and Pfizer, research funding from AstraZeneca, Bayer, Loxo Oncology, Inc. and Puma Biotechnology, and travel, accommodation or expenses from Chugai Pharma and Genentech. ML discloses research funding and advisory board compensation from Loxo Oncology, Inc. and advisory board compensation from Bayer. The Guest Editors received no honorarium for the preparation of the supplement.

## Footnotes

* Note added in proof: The European Medicines Agency granted marketing authorisation for larotrectinib on 23 September 2019 as monotherapy for the treatment of adult and paediatric patients with solid tumours that display a neurotrophic tyrosine receptor kinase (*NTRK*) gene fusion, and who have disease that is locally advanced, metastatic or where surgical resection is likely to result in severe morbidity, and who have no satisfactory treatment options.
